# Praziquantel Facilitates IFN-γ-Producing CD8^+^ T Cells (Tc1) and IL-17-Producing CD8^+^ T Cells (Tc17) Responses to DNA Vaccination in Mice

**DOI:** 10.1371/journal.pone.0025525

**Published:** 2011-10-05

**Authors:** Qiang Zou, Xin Yao, Jin Feng, Zhinan Yin, Richard Flavell, Yanxin Hu, Guoxing Zheng, Jin Jin, Youmin Kang, Bing Wu, Xiaoxuan Liang, Congcong Feng, Hu Liu, Weiyi Li, Xianzheng Wang, Yumei Wen, Bin Wang

**Affiliations:** 1 State Key Laboratory for Agro-Biotechnology, College of Biological Science, China Agricultural University, Beijing, People's Republic of China; 2 Key Laboratory of Medical Molecular Virology of MOH and MOE, Fudan University Shanghai Medical College, Shanghai, People's Republic of China; 3 Center for Infection and Immunity, Institute of Biophysics, Chinese Academy of Sciences, Beijing, People's Republic of China; 4 College of Life Sciences, Nankai University, Tianjin, People's Republic of China; 5 Department of Immunobiology, Yale School of Medicine, New Haven, Conneticut, United States of America; 6 College of Veterinary Medicine, China Agricultural University, Beijing, People's Republic of China; 7 Department of Biomedical Sciences, University of Illinois College of Medicine, Rockford, Illinois, United States of America; University of Nebraska Medical Center, United States of America

## Abstract

**Background:**

CD8^+^ cytotoxic T lymphocytes (CTLs) are crucial for eliminating hepatitis B virus (HBV) infected cells. DNA vaccination, a novel therapeutic strategy for chronic virus infection, has been shown to induce CTL responses. However, accumulated data have shown that CTLs could not be effectively induced by HBV DNA vaccination.

**Methodology/Principal Findings:**

Here, we report that praziquantel (PZQ), an anti-schistoma drug, could act as an adjuvant to overcome the lack of potent CTL responses by HBV DNA vaccination in mice. PZQ in combination with HBV DNA vaccination augmented the induction of CD8^+^ T cell-dependent and HBV-specific delayed hypersensitivity responses (DTH) in C57BL/6 mice. Furthermore, the induced CD8^+^ T cells consisted of both Tc1 and Tc17 subtypes. By using IFN-γ knockout (KO) mice and IL-17 KO mice, both cytokines were found to be involved in the DTH. The relevance of these findings to HBV immunization was established in HBsAg transgenic mice, in which PZQ also augmented the induction of HBV-specific Tc1 and Tc17 cells and resulted in reduction of HBsAg positive hepatocytes. Adoptive transfer experiments further showed that PZQ-primed CD8^+^ T cells from wild type mice, but not the counterpart from IFN-γ KO or IL-17 KO mice, resulted in elimination of HBsAg positive hepatocytes.

**Conclusions/Significance:**

Our results suggest that PZQ is an effective adjuvant to facilitate Tc1 and Tc17 responses to HBV DNA vaccination, inducing broad CD8^+^ T cell-based immunotherapy that breaks tolerance to HBsAg.

## Introduction

Chronic viral hepatitis B, a disease caused by hepatitis B virus (HBV), is a worldwide health problem [1], [2], [3]. The host immune response plays a key role in the outcome of HBV infection [4], [5], [6]. Efficient induction of multi-specific CD8^+^ T cell responses against the core and surface antigens of this virus can control HBV infections [7], [8], [9]. Particularly, reports have described the involvement of two main subsets of CD8^+^ T cells, the IFN-γ-producing Tc1 cells [10], [11] and IL-17-secreting Tc17 cells [12], [13], [14]. It becomes increasingly clear that efficient expansion of virus-specific Tc1 cells resolves HBV infection by cytolytic and noncytolytic mechanisms [15], [16], [17], although the mechanism of action by Tc17 cells remains unclear.

HBV DNA immunization is a promising strategy for inducing strong CD8^+^ T cell-mediated immunity in mice [18], [19], [20], [21], whereas this effect has been only transient and weak in clinical trials [22], [23]. To move beyond various approaches already employed to improve DNA vaccination, we have explored the use of novel adjuvants that would specifically potentiate the Tc1 and/or Tc17 type of T cells as a means to overcome the problem.

Praziquantel (PZQ) has been used for the treatment of *Schistoma japonicum* infection without severe side effects [24]. It has been found that the humoral and cellular immune responses of the host were enhanced after being treated with this drug [25], [26]. We recently demonstrated that PZQ could act as an adjuvant to enhance cellular responses to HBsAg DNA vaccination in mice [27]. However, it was not clear how PZQ affected CD8^+^ T cell responses and whether the effect of PZQ was strong enough to break immune tolerance in HBsAg-transgenic mice. We have begun to address these questions in the present report.

## Results

### PZQ enhanced CD8^+^ T cell-mediated responses to HBV DNA vaccine

**Figure 1:**
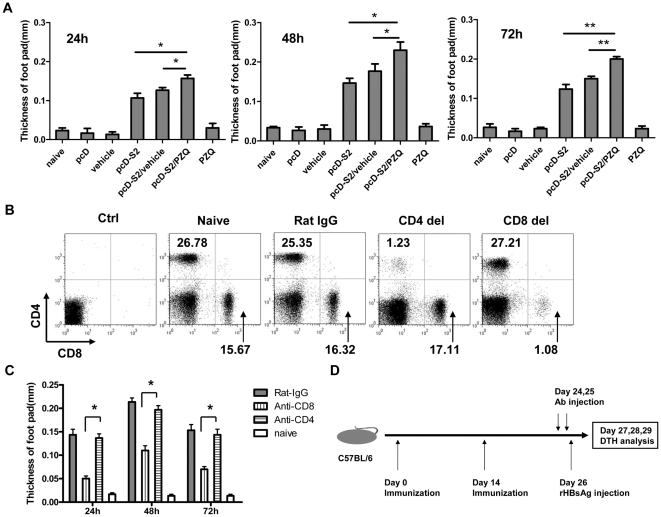
Induction of DTH was mediated by CD8^+^ T cells in pcD-S2 and PZQ immunized group. A: Twelve days after the second immunization with indicated the combinations, all groups were challenged with the rHBsAg in the right foot pad as test and with a saline solution in the left foot pad as control. The thickness of footpads was measured at 24, 48 and 72 h. B, Mice were depleted of CD4^+^ (CD4del) or CD8^+^ (CD8del) T cells by in vivo injection of specific mAbs. The efficiency of depletion was determined by flow cytometry of splenocytes. The numbers indicate the percentages of CD4^+^ or CD8^+^ T cells. C: Ten days after the second immunization of pcD-S2 plus PZQ, mice were injected i.p. with anti-CD4 mAb, anti-CD8 mAb, or rat IgG and challenged 2 days later with rHBsAg. DTH was measured at 24 h. D: The immunization schedule is shown. The data shown summarizes one of three experiments, all of which demonstrated similar results (* p<0.05; ** p<0.01).

Delayed-type hypersensitivity (DTH) is a well-established readout for T cell responses to vaccination [28], [29]. To determine the immunogenic effect of PZQ on HBV DNA vaccination, DTH responses from C57BL/6 mice immunized with pcD-S2 in the presence or absence of PZQ were compared. Using HBsAg as a rechallenging antigen, we found that PZQ augmented the DTH response to HBsAg (Fig. 1A).

To further determine the subpopulations of T cell in the augmented DTH, over 90% of either CD4^+^ or CD8^+^ T cells were depleted with anti-CD4 or anti-CD8 mAbs respectively (Fig. 1B). Interestingly, the depletion of CD8^+^ T cells significantly blunted DTH, whereas the depletion of CD4^+^ T cells had little impact (Fig. 1C–D), suggesting that PZQ preferentially augmented CD8^+^ T cell responses to pcD-S2 vaccination.

### Induction of Tc1 and Tc17 cells during DNA vaccination with PZQ

**Figure 2:**
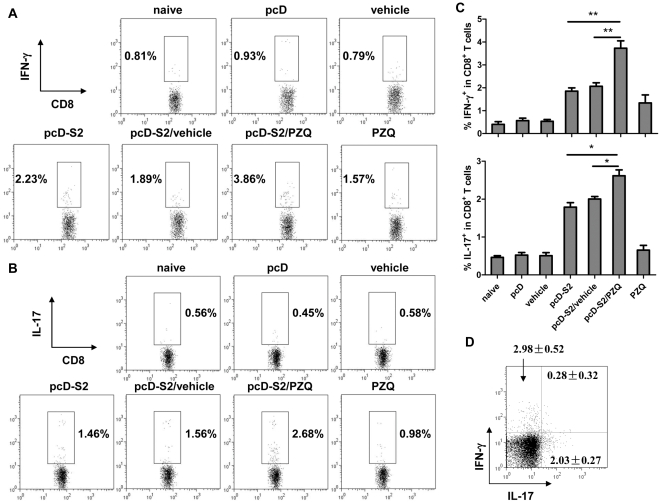
Tc1 and Tc17 cells induction in response to HBsAg DNA vaccination with PZQ. A–B, Splenic T cells were isolated on day 14 after the third immunization and were stimulated with the HBsAg-derived peptide S208-215 in the presence of brefeldin A (5 µg/ml) for 6 h in culture and immunostained for surface CD8, and intracellular IFN-γ and IL-17. C, Summary of the percentage of IFN-γ- and IL-17-expressing CD8^+^ T cells. D, Intracellular staining for IFN-γ and IL-17 in CD8^+^ T cells. C57BL/6 mice were immunized with pcD-S2 and PZQ and CD8^+^ T cells isolated on day 7 after third immunization were stimulated in vitro. Data shown are representative of 3 independent experiments (* p<0.05; ** p<0.01).

Effector cytotoxic CD8^+^ T cells can be divided into IFN-γ-producing (Tc1) and IL-17-producing (Tc17) subtypes [10], [11], [12], both of which might be involved in DTH [30], [31], [32], [33], [34]. To delineate which was induced by PZQ, we isolated splenic cells from mice immunized with pcD-S2, pcD-S2 plus vehicle, or pcD-S2 plus PZQ and restimulated the cells in vitro with an HBsAg-derived peptide, S208-215. The CD8^+^ T cells from mice immunized with pcD-S2 and PZQ secreted both IFN-γ and IL-17 at a higher level than those of the controls (Fig. 2A–C), suggesting PZQ induced both Tc1 and Tc17 subtypes. To identify the cytokine-producing cells, restimulated CD8^+^ T cells were intracellularly stained with anti-IL-17 and IFN-γ mAbs and examined by flow cytometry. As expected, many IFN-γ single-positive (Tc1) and IL-17 single-positive were observed (Tc17), but very few IFN-γ and IL-17 double-positive, CD8^+^ T cells were shown (Fig. 2D).

**Figure 3:**
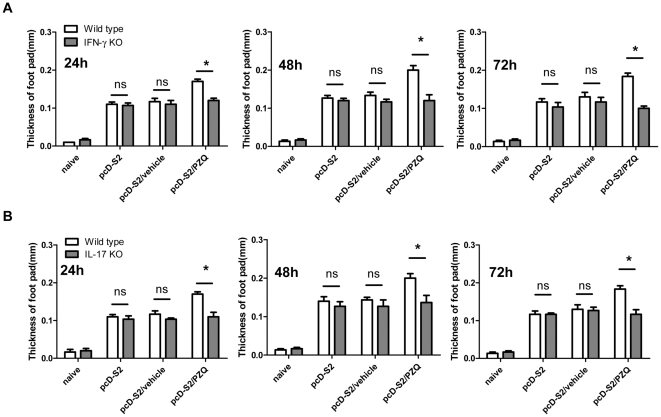
Impairment of DTH in IFN-γ KO mice and IL-17 KO mice immunized with pcD-S2 and PZQ. Mice were challenged with rHBsAg 12 days after the second immunization. DTH was measured at 24, 48 and 72 h. A and B, DTH of IFN-γ and IL-17 KO mice, respectively. Data shown are representative of three independent experiments (* p<0.05; ns, p>0.05).

To determine whether the generation of Tc1 or Tc17 cells was necessary for the augmented HBsAg-specific DTH, pcD-S2 immunization was carried out in IFN-γ KO mice and IL-17 KO mice. Compared to that of wild-type mice, DTH was significantly decreased in the IFN-γ KO or IL-17KO mice (Fig. 3A and 3B). Taken together, PZQ was shown to facilitate both Tc1 and Tc17 cells.

### PZQ broke tolerance to HBsAg in HBsAg-transgenic mice

**Figure 4:**
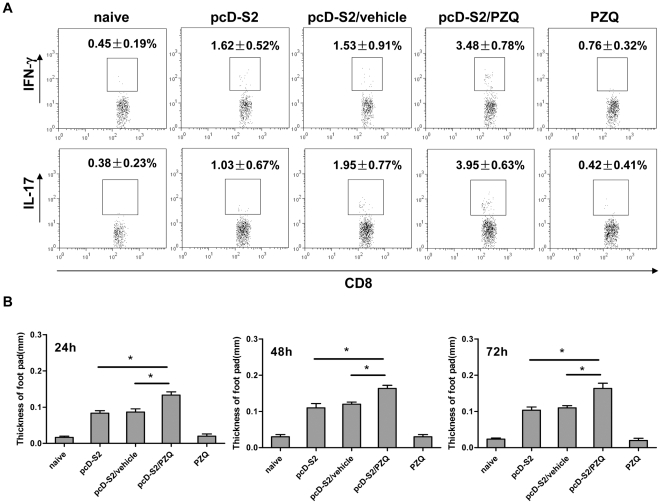
PZQ enhancement of the induction of Tc1 and Tc17 cells in HBsAg-transgenic mice. A, Splenic T cells were isolated on day 14 after the third immunization and stimulated with S208-215 in the presence of brefeldin A (5 µg/ml) for 6 h in culture. CD8^+^ T cells were intracellularly immunostained for IFN-γ and IL-17. B, 12 days after the second immunization, DTH was measured at 24, 48 and 72 h later. Results were representative of three independent experiments (* p<0.05).

**Figure 5:**
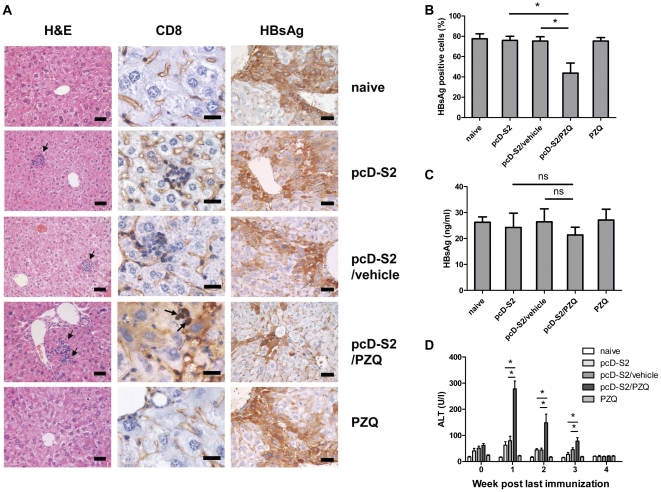
Histopathology of the liver and serology assays. A: livers from each group were obtained on day 14 after the third immunization and fixed, sectioned, and stained with H&E. Bar, 50 µm. CD8-specific immunostaining of the liver from HBsAg transgenic mice on day 14 after final immunization. Bar, 20 µm. Specific immunostaining of HBsAg on day 14 after final immunization. Bar, 50 µm. B: Percentage of HBsAg-positive liver cells in indicated groups on day 14 after final immunization. C: HBsAg antigen in the serum on day 14 after final immunization. D: ALT activity in the serum on weeks 0, 1, 2, 3, and 4 after the third immunization. Results were representative of three independent experiments. There were four mice in each group (* p<0.05; ns, p>0.05).

To test if PZQ-induced Tc1 and Tc17 cells in vivo had a potential to clear tolerized viral antigens, HBsAg-Tg mice were immunized with pcD-S2 in the presence and absence of PZQ. We observed that PZQ also increased the levels of Tc1 and Tc17 cells and the HBsAg-specific DTH in these animals (Fig. 4A and 4B). To assess the possible therapeutic effects, we examined lymphocyte infiltration in the liver of immunized HBsAg-Tg mice. As shown in Fig. 5A, no infiltration was detected in nontreated mice. The highest level of infiltration was noted in mice immunized with pcD-S2 and PZQ, as compared to mice immunized with pcD-S2 alone or with vehicle. In addition, the PZQ-treated DNA vaccine group was the only group showing a significant number of CD8^+^ T cells in the infiltrates, presumably of the Tc1 and Tc17 subtypes (Fig. 5A). No CD4^+^ T cells were detected (data not shown) in this group. Lastly, no obvious change in liver morphology was noted in this group, suggesting that the induced CD8^+^ T cells were not overtly pathogenic.

Consistent with the observed CD8^+^ T cell infiltration, a significant reduction of HBsAg-positive hepatocytes following immunizations with pcD-S2 plus PZQ was observed (Fig. 5A and 5B). However, the level of HBsAg in the blood was not significantly changed (Fig. 5C).

The reduction of HBsAg-positive hepatocytes was likely due to killing of these cells by the infiltrating CD8^+^ T cells. To confirm this, serum ALT levels were analyzed on weeks 0, 1, 2, 3, and 4 after the final immunization. As depicted in Fig. 5D, serum ALT reached the highest level on week 1 and gradually declined to a basal level on week 4 in mice immunized with pcD-S2 plus PZQ. In contrast, ALT levels remained low throughout the same period in control mice immunized with pcD-S2 alone and pcD-S2 plus vehicle (Fig. 5D). This result thus supported our speculation.

### PZQ-induced cytolytic CD8^+^ T cells are effectors in killing of HBsAg-positive hepatocytes

**Figure 6:**
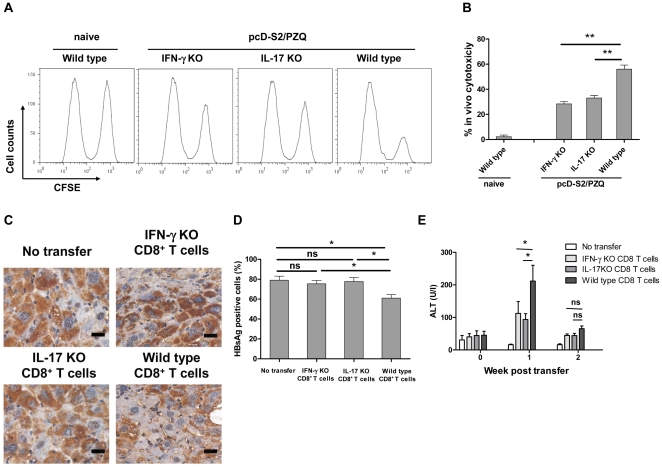
Adoptive transfer of PZQ-induced CD8^+^ T cells. A: In vivo cytotoxic lysis was performed in the wild type, IFN-γ KO and IL-17 KO mice on day 7 after the third immunization with pcD-S2 and PZQ. B: The percentage of specific lysis was summarized. C: HBsAg-specific immunostaining of HBsAg of livers from the HBsAg transgenic mice was analyzed on day 14 after adoptive transfer. Bar, 20 µm. D: Percentage of HBsAg-positive liver cells. E, ALT level in the serum was detected on weeks 0, 1 and 2 after adoptive transfer of CD8^+^ T cells. The data shown are representative of three independent experiments (* p<0.05; ** p<0.01; ns, p>0.05).

In order to further identify the particular role of Tc1 and Tc17 cells in elimination of HBsAg-positive hepatocytes, C57BL/6 wild type, IFN-γ KO, or IL-17 KO mice were immunized with pcD-S2 and PZQ, followed by analysis of HBsAg specific CD8^+^ T cell-mediated killings in vivo. As expected, the highest percentage of antigen-specific killing (∼60%) was noted in immunized wild type mice, as compared to that in IFN-γ KO (∼30%) or IL-17 KO (∼35%) mice (Fig. 6A–B). This result demonstrated that both Tc1 and Tc17 cells were involved in the elimination of HBsAg positive hepatocytes.

To establish the role of these CD8^+^ T cells as the effectors for killing of HBsAg-positive hepatocytes, CD8^+^ T cells were isolated from immunized mice and purified to 90–95% purity on day 7 after the final immunization, and adoptively transferred intravenously into normal HBsAg-Tg mice at 5×10^6^ cells per recipient animal. Only the transfer of CD8^+^ T cells from the wild type mice could significantly reduce HBsAg expression in the liver of the recipients, whereas CD8^+^ T cells from the IFN-γ KO or IL-17 KO mice were not effective (Fig. 6C–D). Consistently, the ALT level rose after adoptive transfer of the wild type CD8^+^ T cells and returned to a normal level on week 2 post transfer (Fig. 6E). These results thus established that both Tc1 and Tc17 cells were the effectors.

## Discussion

HBV-specific CD8^+^ T cells with killing of HBV-infected hepatocytes is the most desirable option for the control of chronic HBV infection [35]. In this report, we find the anti-Schistosoma drug praziquantel (PZQ) to be highly effective at eliciting Tc1 and Tc17 responses in both wild type (WT) and HBsAg-Tg mice. Importantly, the induced HBsAg specific CD8^+^ T cells are potent enough to break established immune tolerance and kill HBsAg positive hepatocytes.

PZQ was previously shown in schistosomiasis patients to significantly alter immune responses [25], [26], [36]. However, it was not explored as an immune adjuvant until its recent demonstration as an augmenting effect on cellular responses to DNA vaccination [27]. As such, we have sought to further characterize this drug in terms of the types of immune responses and subsets of T cells it affects. To assess the effects of PZQ, DTH was utilized. In this study, we demonstrated that PZQ induced a higher DTH response than that of a DNA vaccine alone. Although Th1 and Tc1 cells have been previously studied in detail to show their associations with IFN-γ dependent DTH responses, Th17 cells, Tc17 and macrophages have also been demonstrated to involve the induction of DTH as well [31], [37], [38]. During DNA vaccination alone, the levels of DTH as showed in Fig. 3 had a slight difference between KO and wild type mice, suggesting that DTH could be derived from CMI other than Tc1 and Tc17 cells. In the case of DNA vaccination with PZQ, however, Tc17 and Tc1 were the main contributors for DTH. Therefore, the causes of DTH could be due to a complicated mechanism.

Tc1 cells are the classical cytotoxic T cells that produce IFN-γ and TNF-α and can destroy virally infected cells through the targeted secretion of perforin and granzymes from lytic granules [39], [40], [41]. Tc17 cells, which produce IL-17 [42], [43], [44], [45], [46], [47], are also cytotoxic [12], [42], [48] and have been shown to protect animal against acute influenza virus infection [48]. To date, HBV specific Tc1 cells have been demonstrated to control chronic HBV infection [7], [15] and eliminate HBV infected hepatocytes in transgenic animal model [19], [20]. In contrast, the role of Tc17 cells in HBV protection is not understood. Our data show, for the first time, that Tc17 cells kill HBsAg-positive hepatocytes in vivo. This observation appears to suggest that, similar to Tc1, Tc17 cells are essential effectors.

Although it has been demonstrated that Tc17 cells-mediated cytolytic killing could support the anti-viral immunity through recruiting neutrophils by IL-17 secretion, by secreting IFN-γ, or by expressing FasL [12], [42], [48], our data demonstrated that these IL-17-postive and IFN-γ-negative Tc17 cells were involved in antigen-specific cytotoxic responses that had capacity to kill HBsAg-positive hepatocytes in vivo. These IL-17-producing Tc17 cells mediated the broad CTL mainly through a common cytolytic or Fas-FasL as previous studies indicated [12], [48]. In future experiments, it will be determined if neutrophils are recruited and infiltrated to the sites. As well, the expressions of FasL of Tc17 cells will be analyzed.

The specific role of IL-17 involved in anti-viral immunity remains elusive. It has been shown that blockade of IL-17 resulted in diminishing the survival in response to lethal influenza virus challenge [49], indicating that IL-17 may play some roles in the anti-viral response and protection. IL-17 had been found to participate in host defense against VV infection and recruit neutrophils [50]. Tc17 cells could protect against acute viral infection through recruiting neutrophils or through the secretion of FasL [12], [42], [48]. Perhaps IL-17 could recruit or activate the innate and adaptive immune cells against viral infection. Therefore, its anti-viral role should be further investigated.

T cell-mediated cytolytic killing can result in tissue injury. Liver injury was detected through the elevation of serum ALT, which reached the highest level on week 1 post last immunization in HBsAg-Tg mice and gradually declined to the basal level. However, the extent of liver damage appeared to be limited, as histology of the liver during the same time window revealed no gross change in liver morphology. Therefore, specific killing of the HBsAg positive cells may cause limited damage to the liver, but not result in gross pathology. Further investigation is needed to further clarify this point.

Elimination of HBsAg positive hepatocytes did not significantly change the level of HBsAg in serum. This disconnection has been described in several previous publications. Particularly, it was reported that therapeutic vaccination cleared chronic hepatitis B virus and caused HBeAg seroconversion in chronic carrier chimpanzees, with HBsAg remaining in serum throughout the observation period [51]. Another report showed that the levels of serum HBV DNA and HBeAg were decreased without HBsAg seroconversion in patients [52]. Therefore, one could speculate that a small proportion of HBsAg positive hepatocytes could maintain the high concentration of HBsAg in the serum, resulting in a mild decrease or clearance of HBsAg in the serum, even if a large proportion of HBsAg positive cells are already cleared.

In summary, our results demonstrate ,for the first time, that a HBsAg DNA vaccine, with PZQ, used as adjuvant can induce both Tc1 and Tc17 in vivo and result in elimination of HBsAg positive cells. Since PZQ is already an approved clinic drug, its use for HBsAg DNA vaccination should be safe enough to advance to clinical trials to further examine its potential as a human adjuvant. A more general application of PZQ as a pro-Tc1 and Tc17 adjuvant may also be explored for other immunotherapeutic vaccines to control other chronic viral infections.

## Materials and Methods

### Animals and reagents

Female C57BL/6 mice of 8–10 weeks of age were purchased from the Animal Institute of Chinese Medical Academy (Beijing, China). HBsAg-transgenic (Tg) mice (C57BL/6J-Tg(Alb1HBV)44Bri/J) and IFN-γ KO (B6.129S7-Ifng^tm1Ts^/J) mice were purchased from the Jackson Laboratory (Bar Harbor, Maine). IL-17 KO mice (C57BL/6 background) were kindly provided by Richard Flavell (Yale University School of Medicine, New Haven, CT). All animal protocols (#28482) were approved by the Animal Welfare Committee of China Agricultural University and housed with pathogen-free food and water under 12 h light-cycle conditions.

Praziquantel (NCPC, Hebei, China) was initially dissolved in ethanol to 6.7% and subsequently diluted to 0.5% with 0.9% saline solution. The vehicle was 7.5% ethanol with 0.9% saline solution. CHO cells expressing recombinant HBsAg (rHBsAg) was kindly provided by China North Pharmaceutical Group Corporation (NCPC, Hebei, China). The HBsAg-derived peptides S208-215 (ILSPFLPL; H-2K^b^-restricted) were synthesized by GL BiochemCo., Ltd. (Shanghai, China). Rat anti-mouse CD4 mAb (GK1.5), Rat anti-mouse CD8 mAb (53-6.7) and rat IgG were purchased from eBioscience (San Diego, CA, USA). Fluorescent-labeled anti-mouse mAbs including anti-CD4-FITC, anti-CD8-FITC, anti-CD8-APC, anti-IFN-γ-PE, anti- IL-17-PE and anti-IL-17-APC were purchased from BD PharMingen (San Diego, CA, USA). The anti-HBsAg antibody for immunohistochemical staining was produced in C57BL/6 mice immunized 3 times with rHBsAg and alum as adjuvant.

### Plasmid construction and preparation

For mouse studies, the HBV DNA vaccine (pcD-S2) was prepared as described previously [27]. The plasmid was maxi-prepared by the alkaline method, subsequently purified by Qiagen Maxi prep kit (Qiagen Inc., Duesseldorf, Germany), and diluted in saline solution.

### Immunization

Mice were randomly divided into six or seven groups (n = 6 each) according to the design of different experiments, and were immunized with 100 µg pcD-S2 per mouse [27]. pcD was the empty vector for pcDNA3. All mice were immunized on day 0 and boosted on day 14.

### Elicitation of delayed-type of hypersensitivity (DTH)

Twelve days after the second immunization, all groups were challenged with 10 µg of rHBsAg in the right footpad as test and saline solution at the left footpad as control. The thicknesses of footpads were measured at 24, 48 and 72 h with a micrometer and calculated using the following formula: thickness footpad = thickness of right footpad - thickness of left footpad.

For elimination of CD4^+^ and CD8^+^ T cells, anti-CD4 mAb (200 µg/mouse), anti-CD8 mAb (200 µg/mouse), or rat IgG (200 µg/mouse) was injected (i.p.) twice into immunized mice on days 10 and 11 after the second immunization. The mice were challenged on day12 and DTH was measured as described above.

### Flow cytometry

Splenic cells were isolated on day 7 after the final immunization and stimulated with 10 µg/ml S208-215 in the presence of brefeldin A (5 µg/ml) for 6 h at 37°C and 5% CO_2_. Collected cells were fixed with 4% paraformaldehyde and permeabilized with 0.1% saponin (Sigma-Aldrich). For immunostaining of cytoplasmic IL-17 and IFN-γ, and surface CD8, the appropriate concentrations of fluorescently labeled anti-mouse monoclonal antibodies were added to permeabilized cells for 30 min on ice followed by washing twice with cold PBS. Samples were analyzed by a FACSCalibur.

### Histopathology

On day 7 after final immunization, liver tissue was paraffin-embedded, sectioned, stained with H&E, and immunostained with anti-HBsAg mAb, anti-CD4 mAb, or anti-CD8 mAb. Analysis was performed under a light microscope for histological changes.

### In vivo cytotoxic assay

Splenocytes from naïve C57BL/6 mice were pulsed with 10^−6^ M HBsAg-derived peptides S208-215 and labeled with a high concentration of CFSE (15 µM, CFSE^high^ cells) as target cells. A portion of the same splenocytes was labeled with a low concentration of CFSE (0.5 µM, CFSE^low^ cells) without peptide pulse as a non-target control. The target and control cells were mixed in a 1∶1 ratio and injected into immunized mice at 2×10^7^ total cells per mouse via the tail vein on day 7 after the third immunization. Eight hours later lymphnodes and the spleen of injected mice were removed and the target and control cells were analyzed by their differential CFSE fluorescent intensities using a FACSCalibur (BD Biosciences, USA). Specific lysis was calculated using the following formula: Percentage specific lysis = [1−(ratio unprimed/ratio primed)×100], where ratio = percentage CFSE^low^/percentage CFSE^high^.

### Adoptive transfer of CD8^+^ T cells

On day 7 after the third immnuzation with pcD-S2 and PZQ, single-splenocyte suspensions were prepared from spleen of wild type, IFN-γ KO or IL-17KO mice. CD8^+^ T cells were isolated and purified using the MagCellect Mouse CD8^+^ T Cell Isolation Kit according to the manufacturer's protocol (R&D Systems, Inc., Minneapolis, USA). Purity of each cell preparation was 90–95%. The cells were adoptively transferred intravenously into normal HBsAg transgenic mice at 5×10^6^ per recipient mouse.

### Statistical analysis

Pairwise differences were analyzed by the two-sided Student's *t* test. For multi-group analysis, ANOVA and the Bonferroni test were used. A value of *p*<0.05 was considered to be statistically significant.
